# The Role of Videotaped Clinical Skills Aggregated Peer Evaluations in the Enhancement of Evaluation Skills of Individual Medical School Faculty Members

**DOI:** 10.7759/cureus.37871

**Published:** 2023-04-20

**Authors:** Munevver Duran, Joseph M Hendrix

**Affiliations:** 1 Medical School, University of Texas Southwestern Medical School, Dallas, USA; 2 Anesthesiology, University of Texas Southwestern Medical School, Dallas, USA

**Keywords:** instruction improvement, faculty assessment, clinical skill teaching, fourth-year medical student education, medical education

## Abstract

The conventional methodology for appraising medical students has some limitations like inherent subjectivity, unstructured nature, and bias. Implementing the Objective Structured Clinical Examination (OSCE) can mitigate these shortcomings. However, the OSCE presents challenges, including substantial financial costs and time-intensive processes, particularly when assessing a large cohort of students. Consequently, an alternative assessment was needed to keep the advantages of OSCE and mask its limitations, especially in resource-constrained settings. In addition, many scholars have expressed concern over medical students' and interns' inadequate interviewing and physical examination competencies in recent years. Due to easy availability, videotaping is a convenient method for objectively observing students for aggregate review by faculty in order to ascertain what exactly faculty are assessing during medical student evaluations. This technique allows for aggregate faculty group improvement in the ability of educators to assess students' technical proficiency, data collection capabilities, standardized patient interaction demeanor, and strategies for fostering standardized patient comfort. Nonetheless, aggregate evaluation of videotape recordings for faculty assessment development or reliance on verbal feedback from medical students about faculty's ability to assess student skill is a matter of debate. Due to increased subspecialization, the subspecialist or specialist examiners face difficulties in assessing students' skills in specific and/or generalized domains. Despite these challenges, assessing the ability of faculty members' observation and subsequent evaluation of medical trainees remains a vital aspect of assessment throughout various specialties. This paper presents the concept of faculty members individually observing and rating premade recordings of standardized students performing clinical skills for evaluation so that when the individual faculty members' ratings are aggregated and summarized collectively for evaluation by the faculty members as a group, both the group and the individual faculty members will gain a greater understanding of what are appropriate ratings versus outlier ratings.

## Introduction and background

The general concept of the traditional system of evaluating medical students could be criticized as being subjective, non-structured, and biased. These drawbacks would be avoided if the objective structured clinical examination (OSCE) was used. The OSCE, on the other hand, is expensive and time-consuming when examining a large number of students. Numerous authors have expressed concern regarding medical students' and interns' subpar interviewing and physical examination skills in recent years [1-5]. Edelstein and Ruder noted the general perception that physician interviewing skills are lacking among medical students and house staff [1]. In the undergraduate teaching of psychiatry, videotaped recordings are widely used to teach interview procedures and show phenomenology and treatment [2]. Being easily available, videotape is a feasible method for direct student observation. This allows educators to collect educational data and evaluate students' technical skills and their approach toward patients and examination skills [3]. However, it is unknown if videotape recording is better than verbal feedback to evaluate the clinical skills of medical students. In addition, as the medical practice becomes more subspecialized, clinical examiners may lack specific domain competence in every field and may not be equipped to appraise the current practice standards or student experiences in many subspecialties. Despite standardized checklists, the question remains as to whether each individual faculty member appreciates what they are observing as well as how their ability to rate students regardless of standardized checklists [4]. However, faculty observation of medical trainees is an essential component of assessment across specialties [5]. The intent of this review is to provide information about how faculty viewing and rating of premade recordings of medical students performing clinical skills for evaluation will allow, once the ratings are aggregated and summarized for a faculty group, both the faculty group and the individual faculty member to understand where the group average and range of ratings lie such that each individual faculty member will have an opportunity for growth and development insofar as where their ability to rate students' clinical skills is compared to their peers.

In a study by Fenton and O' Gorman, 60 final-year medical students were evaluated in psychiatry “using three methods: a multiple-choice questionnaire based on a series of short videotaped interviews with psychiatric patients, an examination of a traditional long 'case' with the presentation of the history, mental state findings, and formulation about diagnosis and management to a panel of three examiners; and a traditional oral examination about the principles and practice of psychiatry” [2]. In another study, a statistical examination of the distribution of the correct answers to videotaped questions revealed that the students excelled at identifying mental state symptoms and signs and selecting the proper diagnosis [6]. This study was conducted to determine whether the findings of a student's evaluation during an OSCE were comparable to the results of an evaluation utilizing a video recording of the same OSCE. The results indicated that evaluating a student's clinical skills using a video recording is as reliable as the more conventional live evaluation. OSCE videos help teach clinical skills and preparation for OSCEs. The quantity of OSCE videos viewed by students was moderately related to their self-efficacy and OSCE readiness [7]. This study validates the positive impact of OSCE videos on student clinical competence acquisition. The ability of this self-insight and viewing of one's own performance is likely applicable to individual faculty members' abilities to assess student clinical skill performance. The full process of history taking and physical examination and more importantly the ability to assess the competency of students' performance of the process is integral to medical education. To help faculty better use these materials, institutions should train their faculty on how to assess students' abilities, use interactive technology to improve faculty-student communication, and encourage using smart devices to increase accessibility.

The most effective use of videotaped interviews is to show a variety of clinical situations that allow applicants to engage in debate and educated conjecture regarding the mental states being portrayed [2]. “Observational evaluation provides continuing data on trainee performance with actual patients, and good assessment assists medical educators in meeting their professional commitment to properly self-regulate”[5]. Direct observation is crucial for teaching medical students general clinical skills and developing standardized patient situations. Conveniently, video provides for such an evaluation of physical examination and conversational skills. It also provides insight into the student's clinical thinking ability. In addition, educators can pause, replay, and adjust the speed and volume of the videos to obtain a better analysis of the medical students' performance during their exams. Hence, it lowers the chances of missing parts of the interaction and allows teachers to pinpoint specific attributes of the clinical exam when giving feedback. Overall, direct observation of medical students through recordings facilitates the evaluation of their competency, encourages preferred behaviors, and affords early opportunities to correct mistakes. Regardless, residents and medical students report that being monitored during clinical rotations is uncommon despite these benefits.

The Department of Radiodiagnosis and the Television Service at the University of Leeds [8] worked together to make a set of six videotapes that explain several aspects of chest radiology using only radiographs. A small group of students in their last year of medical school watched these tapes over a few days. Afterward, their scores on factual questions were compared to how they felt about the learning experience, which they reported anonymously and without knowing their scores. Students with the best grades were usually the most critical of the videotapes. Students who got lower grades were more likely to say positive things about the tapes when they scored well, and negative things when they did not. The students used their scales to rate the experience, which led to a wide range of detailed and helpful suggestions. In general, the students were pleased with how well videotapes could show fine radiographic details and consequently concluded that they are a suitable medium for knowledge assessment.

In a case study by Mitchell et al., which aimed to improve OSCE feedback through an analysis of student reflection on their recorded video performances, students were significantly better at self-assessing their clinical performance after viewing their videos of their OSCE stations in comparison to their self-assessment right after their performance [9-10]. Furthermore, using a video in self-assessment led to a significant rise in the word count of the medical students' reflections, providing them with more specific, personalized learning goals to improve their clinical skills. This study further demonstrated that using videos to assess OSCE performance could increase the accuracy and functionality of the feedback process by increasing student self-efficacy in formal educational settings [10].

## Review

With the current focus on building an outcomes-based medical education system that increases trainee growth and patient safety, robust work-based evaluation systems are in considerable demand. Among the vast array of diagnostic tools, OSCE is one viable option. However, while objective structured clinical examinations have become the standard for evaluating clinical competence in a training setting, they are not applicable in all situations [9].

Although OSCE is a widely used measure to assess the clinical skills of medical students, highlighting the importance of why educators should continue to research and develop enhancement methods for its grading, it also has many fundamental limitations. According to the international multi-center qualitative study conducted by Hyde et al., there are many concerns regarding the feasibility and accuracy of the OSCE from both the examiner and the examinee perspectives [11]. Both participants and raters outline the lack of authenticity of OSCE examinations, specifically in their inaccuracy in representing natural clinical environments. Advanced clinical skills such as problem-solving and establishing rapport with various patients are ignored in grading. The examiners reported dissatisfaction due to the lengthy checkbox format and also felt that even good grades do not guarantee that the students acquired skills. This study highlights some of the shortcomings of using the OSCE to evaluate how students will perform in real-life settings as clinicians and the overall viability of the exam. These are essential considerations to consider in educational settings for future improvements [11]. There is, thus, a need to develop a more objective method for the rating and evaluation of students' skills.

On the other hand, the OSCE is an excellent tool for validating professional competence. It can also assess collective participants in the activity. Evaluating and assessing students' abilities and faculty members' abilities to evaluate and assess is critical to ensure fairness among all medical institutions. The OSCE can be a valuable tool for conducting these evaluations to guide their development and create a plan for improvements based on their weaknesses.

As part of the educational relationship that responsible institutions must strive to achieve with their students, ensuring that passing requirements are fundamentally fair amongst medical schools is essential. Individually, evaluators are susceptible to “biases such as leniency, inconsistency, and the halo effect,” contributing to variability [11]. For example, previous research has shown that evaluators from various United Kingdom (UK) medical schools may require profoundly different passing standards for identical OSCE stations. It's possible that badly organized or written checklists can cause a bias effect on a specific site when evaluators from all over the UK watch videotaped performances of clinical skills by students with standardized patients and students. However, this was taken into account by assigning pre-determined levels (clear fail, borderline, clear pass, good) for the performances [12]. This recent study by Sam et al., which aimed to determine the level of inter-rater agreement across assessors in the UK while grading simulated candidate results from an examination station, discovered that assessors' inter-rater agreement was excellent [12]. These findings show a high level of correspondence across the ratings done by various educators for the same simulated performances, supporting standardization.

Ideally, there should be a unique, unbiased, and objective method of examination to assess the clinical skills of medical students. Student clinical examinations should be based on transparent, comparable criteria throughout medical institutions; however, examiner conduct, instruction, and other regional characteristics significantly contribute to differences in school performance [13]. Assessor bias may also affect examination results and, in most studies, examiner variation is the single most significant component variable. Donohoe et al. conducted a large-scale study to determine the role of clinical experience and subject matter expertise of assessors during an OSCE focused on surgical procedures [4]. The multivariate linear regression study found no significant correlation between score dispersion and either a surgeon or sub-specialist experience or consultant versus trainee levels of clinical competency. While there is recent evidence to support the potential standardization of medical students and faculty members' rating of video clinical performances, limitations and biases must be considered in the evaluation and minimized as much as possible.

For large-scale performance assessments in clinical education, it is crucial to ensure that examiners' judgments are consistent among groups of examiners to improve fairness and reassure the public. Standardization ensures the public that all graduates have met predetermined assessment requirements, a crucial element of the educational contract between students and institutions. Video-based Examiner Score Comparison and Adjustment (VESCA) employs video scoring to connect previously unconnected groups of examiners. This link allows for comparing the influence of various examiner groups within a shared frame of reference and the provision of modified "fair" results to students. Despite reliability, the examiner cohort experienced by students significantly influenced their scores, underlining the importance of adequate sampling and examiner training. VESCA's creation and validation may provide a method for measuring and adjusting for potential systematic disparities in scoring patterns between locations in dispersed or countrywide OSCE tests, ensuring comparability and fairness [14].

A growing body of research demonstrates video technology's usage and efficacy in education. For example, the Best Foot Forward Project, a recent Harvard University Center for Education Policy Research study, investigated the use of video classroom observation technologies in the feedback process. According to their preliminary findings, there are various advantages to adopting video technology in the classroom [15]. First, classroom observation videos promote best practices and lessons learned. Video observation technology speeds up instructor exchange and cooperation. The instruction of faculty members utilizing premade video recordings allows the instructor to provide guidance in real time during the viewing of the premade clinical skills performance. The utilization of premade video recordings promotes a non-judgemental and collaborative environment for group instruction of faculty members regarding clinical skills evaluation. This encourages transparency and the transfer of a plethora of knowledge.

Second, videos improve classroom observation, self-efficacy, and agency. Educators have greater control over classroom observation when employing videos as a tool. Faculty member instructors would be unable to provide real-time in-person guidance without biasing or adversely affecting the assessment of a medical student's clinical skills performance. However, utilizing premade video recordings allows for faculty member instruction to avoid any possibility of biasing or adversely affecting an actual student's clinical skills performance in person.

Third, video technology fosters reflective practice and self-assessment among educators. By providing an opportunity to review their own teaching methods and interactions with students, teachers can identify areas for improvement and implement necessary changes in their instructional approach. This self-assessment process fosters professional growth and encourages educators to become more deliberate and thoughtful practitioners.

In the context of medical education and OSCE evaluations, video technology has the potential to significantly enhance the assessment process. Utilizing video recordings of student performances during OSCEs can help minimize examiner biases and increase inter-rater reliability. Furthermore, video-based assessments provide an opportunity for a more in-depth analysis of student performance, enabling evaluators to identify specific strengths and weaknesses that may not be apparent in traditional OSCE settings. This can lead to targeted feedback and individualized learning plans for medical students, ultimately improving their clinical competence and patient care.

Additionally, incorporating video technology in OSCE evaluations can contribute to standardizing assessment criteria across institutions. By facilitating the exchange of videos between different medical schools, it is possible to establish a shared understanding of the expected performance levels and create a more equitable assessment system. This standardization ensures fairness for medical students and reassures the public and healthcare institutions that all graduates have demonstrated the clinical skills to provide high-quality patient care.

In conclusion, the OSCE is a valuable tool for assessing medical students' clinical competence. However, recognizing its limitations and striving for continuous improvement by incorporating innovative methods and technologies, such as video-based assessments, is vital. These enhancements can help mitigate biases, increase inter-rater reliability, and promote standardization of assessment criteria across institutions, ultimately improving the quality of medical education and patient care outcomes. Furthermore, as the field of medical education evolves, it is essential to continue researching and developing best practices to ensure that future physicians are well-equipped to meet the challenges of an ever-changing healthcare landscape.

## Conclusions

The OSCE scores of students from various medical schools should not be directly compared without considering systematic differences. VESCA is a promising method for improving the validity and fairness of distributed OSCEs or national exams. Additionally, internet-based scoring could improve VESCA's viability. The development and validation of VESCA may provide a method for measuring and adjusting for potential systematic disparities in scoring patterns between locations in dispersed or national OSCE tests, hence maintaining parity. VESCA is a slowly growing tool that can enhance medical students' educational experience and training while providing a practical and easily controlled platform and allowing routine measurement of examiner-cohort effects in large-scale OSCE grading systems. Nonetheless, the medical community should continue discussing the quality of the scoring system to establish an equitable and standardized grading system to enhance medical education.
